# Prospects of pathogen whole-genome sequencing in outbreak tracing and antimicrobial resistance surveillance of spinal infections

**DOI:** 10.3389/fcimb.2026.1977484

**Published:** 2026-10-06

**Authors:** Xingsheng Yu, Zhen Jia, Jianhua Li, Zhaohu Mao, Zhengqi Chang

**Affiliations:** 1 Department of Orthopaedics, The 960th Hospital of People’s Liberation Army of China (PLA), Jinan, China; 2 Department of Orthopaedics, The 961th Hospital of People’s Liberation Army of China (PLA), Qiqihar, China

**Keywords:** antimicrobial resistance surveillance, genomic epidemiology, outbreak tracing, spinal infection, whole-genome

## Abstract

Spinal infections are a serious category of deep-seated infectious diseases that pose significant threats to human health. They are caused by a broad and complex spectrum of pathogens, including bacteria, fungi, and rare opportunistic organisms. Traditional diagnostic methods often face limitations in timeliness, accuracy, and source-tracing capability. Whole-genome sequencing (WGS), with its high resolution and throughput, provides comprehensive genetic information from pathogens, opening new avenues for precise diagnosis, outbreak tracing, and antimicrobial resistance monitoring and genomic epidemiology research in spinal infections., and enabling reliable identification of rare and fastidious pathogens that are difficult to characterize by conventional assays. This review systematically summarizes current applications of WGS in pathogen identification, clonal transmission inference, resistance gene and virulence factor analysis, and outbreak investigation. It further discusses the technical advantages, application challenges, and future development directions of WGS in outbreak tracing and resistance surveillance. Ultimately, this review aims to offer novel insights and a theoretical foundation for the precise prevention, control, and clinical management of spinal infections.

## 1 Introduction

Spinal infections—including discitis, vertebral osteomyelitis, and spinal epidural abscesses—represent a significant and growing clinical challenge worldwide. Their incidence has been steadily rising, driven by an aging population, an increasing prevalence of comorbidities such as diabetes mellitus, greater use of spinal instrumentation, and intravenous drug use. These infections are associated with substantial morbidity and mortality; reported mortality rates range from 10% to nearly 20% across cohorts, and delayed diagnosis or treatment carries a high risk of permanent neurological deficits. The clinical presentation is often insidious and nonspecific, with back pain, fever, and neurological deficits appearing in varying combinations. Such nonspecificity frequently delays diagnosis, leading to worse outcomes, including sepsis, neurological impairment, and death. The complexity of spinal infections is further compounded by the diversity of causative pathogens—from common bacteria such as Staphylococcus aureus and gram-negative bacilli to less common organisms, including Mycobacterium tuberculosis, fungi such as Aspergillus species, and rare opportunistic pathogens. This broad pathogen spectrum poses significant challenges for clinical diagnosis and effective antimicrobial therapy.

The pathogen spectrum in spinal infections is notably complex. Staphylococcus aureus remains the predominant pathogen, accounting for over half of spinal epidural abscess cases, and includes both methicillin-resistant and methicillin-susceptible strains with distinct therapeutic implications. Gram-negative bacilli—including Escherichia coli and other Enterobacteriaceae—are also implicated, particularly in healthcare-associated infections and in patients with comorbidities (Zou et al., 2025). Mycobacterium tuberculosis is a well-recognized cause of spinal infections, often presenting as a chronic disease with characteristic cold abscess formation and vertebral destruction, necessitating prolonged antituberculous therapy and, in some cases, surgical intervention (Le et al., 2025). Fungal infections, although rare—such as Aspergillus spinal epidural abscess—can mimic tuberculosis and require a high index of suspicion for timely diagnosis and management (Rule et al., 2021). Moreover, emerging reports highlight the involvement of unusual pathogens, including Streptococcus intermedius and Haemophilus influenzae, underscoring the need for comprehensive microbiological evaluation. The polymicrobial nature of these infections, together with the presence of opportunistic and resistant organisms, complicates empirical treatment strategies and emphasizes the importance of precise pathogen identification.It should be noted that several cited references describe epidemiological data for general osteomyelitis rather than spinal-infection-specific cohorts; large-scale dedicated epidemiological statistics for certain pathogens isolated exclusively from human spinal infections remain limited.

In recent years, advances in high-throughput sequencing technologies and bioinformatics have revolutionized the study of infectious diseases, enabling whole-genome sequencing (WGS) of pathogens directly from clinical samples. WGS provides unparalleled resolution in characterizing pathogen genomes, allowing detailed analysis of genetic diversity, virulence factors, and antimicrobial resistance determinants. The decreasing cost and increasing accessibility of WGS have facilitated its transition from a primarily research tool to a clinical application in infectious disease diagnostics and epidemiology. In the context of infectious disease outbreaks, WGS enables precise outbreak investigation, source tracking, and transmission mapping at single-nucleotide resolution, which is critical for infection control and public health interventions. Furthermore, WGS allows comprehensive surveillance of antimicrobial resistance gene dissemination, informing antimicrobial stewardship and guiding therapeutic decisions. These capabilities are particularly valuable in managing complex infections with diverse and evolving pathogen populations.

Applying whole-genome sequencing to spinal infections offers unique advantages. The ability to obtain complete genetic information from causative pathogens facilitates accurate species identification, detection of resistance genes, and characterization of virulence factors, which are often challenging with conventional culture-based methods due to prior antibiotic exposure or fastidious organisms. WGS enables high-resolution phylogenetic analyses to elucidate evolutionary relationships among strains, thereby reconstructing transmission chains and outbreak sources with precision (Araujo et al., 2024). This molecular epidemiology approach is critical in healthcare settings to identify nosocomial transmission events and implement targeted infection control measures. Moreover, WGS can detect genetic markers associated with antimicrobial resistance, allowing early recognition of resistant strains and facilitating personalized antimicrobial therapy (Fayad et al., 2025). In spinal infections, where delayed or inappropriate treatment can lead to devastating outcomes, such molecular insights can significantly improve patient management and prognosis.

Despite these promising applications, challenges remain in integrating WGS into routine clinical practice for spinal infections. Technical limitations include the need for sufficient pathogen DNA from often paucibacillary samples, potential contamination, and the complexity of bioinformatics analyses requiring specialized expertise (Zhao et al., 2023). Interpretation of genomic data demands robust databases and standardized pipelines to accurately predict resistance phenotypes and virulence potential. Additionally, the clinical utility of WGS depends on timely turnaround to influence therapeutic decisions, which necessitates streamlined workflows and integration with clinical microbiology laboratories (Zhao et al., 2023). Ethical and data privacy considerations also arise with genomic data handling. Nevertheless, ongoing technological advancements and decreasing costs are progressively addressing these barriers, paving the way for broader adoption.

In summary, spinal infections represent a significant and increasing healthcare burden characterized by complex pathogen spectra and challenging clinical management. Whole-genome sequencing has emerged as a powerful tool that can enhance pathogen identification, outbreak investigation, and antimicrobial resistance surveillance in spinal infections. By providing detailed molecular insights, WGS holds the potential to transform the diagnosis, treatment, and prevention of spinal infections, ultimately improving patient outcomes. This review will systematically explore the principles of WGS technology, its current applications in outbreak source tracing and resistance monitoring in spinal infections, the core advantages it offers over traditional methods, existing challenges to its clinical implementation, and future prospects for its integration into precision infectious disease management (**Figure 1**).

**Figure 1:**
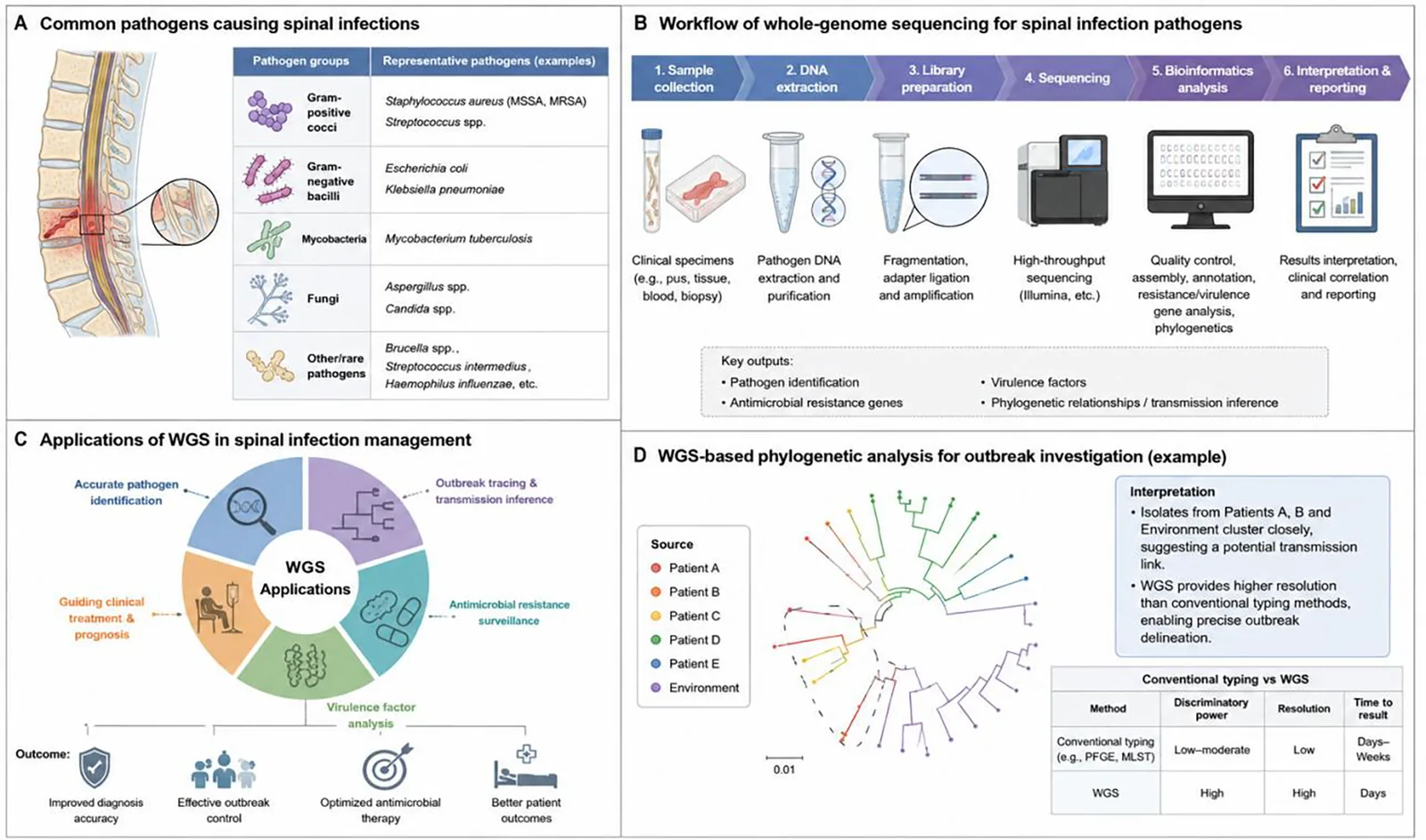
Overview of WGS in spinal infection management, including pathogen spectrum, sequencing workflow, major applications, and phylogenetic outbreak analysis. (**A**: Common pathogens causing spinal infections; **B**: Workflow of whole-genome sequencing for spinal infection pathogens; **C**: Applications of WGS in spinal infection management; **D**: WGS-based phylogenetic analysis for outbreak investigation).

## 2 Basic applications: whole genome sequencing technology in the etiological diagnosis of spinal infections

### 2.1 Diversity of pathogens in spinal infections and limitations of traditional diagnostics

Spinal infections encompass a broad spectrum of causative pathogens, including pyogenic bacteria such as Staphylococcus aureus and Streptococcus species, mycobacteria such as Mycobacterium tuberculosis, fungi such as Histoplasma capsulatum, oomycetes such as Pythium insidiosum, as well as various opportunistic environmental pathogens. This extensive pathogen diversity complicates empirical treatment strategies, often resulting in suboptimal clinical outcomes. Traditional diagnostic methods primarily rely on culture and serological assays; however, these approaches suffer from significant limitations. Culture positivity rates are notably low, especially in patients who have received prior antibiotic therapy, and the prolonged time required for culture results delays timely diagnosis and targeted treatment. Furthermore, rare pathogens such as Pythium insidiosum exhibit extremely slow growth and stringent nutritional requirements under conventional culture conditions, rendering their detection challenging. Even advanced identification techniques such as matrix-assisted laser desorption/ionization time-of-flight mass spectrometry (MALDI-TOF MS) may fail to accurately identify such rare or atypical organisms, leading to frequent misdiagnoses or missed diagnoses—for example, Mangrovibacter phragmitis was initially misidentified as part of the Enterobacter cloacae complex. These diagnostic gaps underscore the urgent need for more sensitive, rapid, and comprehensive pathogen detection methods to improve clinical management of spinal infections (Sivertsen et al., 2022; Liu et al., 2025; Ibrahim et al., 2026; Li et al., 2026).

### 2.2 Technical principles and workflow of whole genome sequencing

Whole-genome sequencing (WGS) of pathogens involves a multi-step process that begins with DNA extraction either from pure cultures or directly from clinical specimens, followed by library preparation, high-throughput sequencing, and comprehensive bioinformatics analysis. The sequencing strategies are broadly categorized into whole-genome shotgun sequencing and whole-genome resequencing. Shotgun sequencing is particularly suited for *de novo* assembly and gene discovery in novel or uncharacterized pathogens, enabling the identification of previously uncharacterized genetic elements. In contrast, resequencing targets known reference genomes to detect genetic variations, including single nucleotide polymorphisms (SNPs) and structural variants, facilitating comparative genomic analyses and epidemiological investigations. The bioinformatics pipeline encompasses quality control of raw sequence data, genome assembly, gene annotation, multilocus sequence typing (MLST), core genome SNP (cgSNP) analysis, and prediction of antimicrobial resistance (AMR) genes and virulence factors. This comprehensive genomic characterization provides a robust foundation for pathogen identification, outbreak source tracing, and monitoring of resistance gene dissemination, thereby supporting precision medicine approaches in spinal infection management (Tang et al., 2025; Douan et al., 2026; Li et al., 2026).

### 2.3 Technological evolution from pathogen identification to genomic epidemiology

The evolution of WGS technology has transformed the landscape of infectious disease diagnostics and epidemiology. Initially applied to sequencing single isolates for pathogen identification, WGS has advanced to enable multi-strain comparative genomics, facilitating detailed phylogenetic and population structure analyses. This progression has allowed subspecies-level resolution, such as resolving Streptococcus agalactiae strains into serotype III-ST283 clones, which elucidates differences in virulence and transmission dynamics. It should be emphasized that WGS is mostly performed on pure cultured isolates, whereas metagenomic next-generation sequencing (mNGS) directly recovers nucleic acids of all microorganisms from primary clinical specimens. The two techniques are complementary rather than interchangeable for spinal infection diagnostics. The integration of WGS with metagenomic next-generation sequencing (mNGS) further expands its utility by enabling direct pathogen genome recovery from clinical samples without the need for culture. This is particularly advantageous for detecting rare or fastidious organisms in spinal infections, where obtaining pure cultures is often challenging. Such genomic epidemiology approaches provide precise pathogen identification, resistance profiling, and outbreak investigation capabilities, enhancing infection control and guiding targeted therapy. The convergence of WGS and mNGS thus represents a paradigm shift in spinal infection diagnostics, offering unprecedented resolution and speed in pathogen characterization (Tang et al., 2025; Douan et al., 2026; Ibrahim et al., 2026; Li et al., 2026) (**Figure 2**).

**Figure 2:**
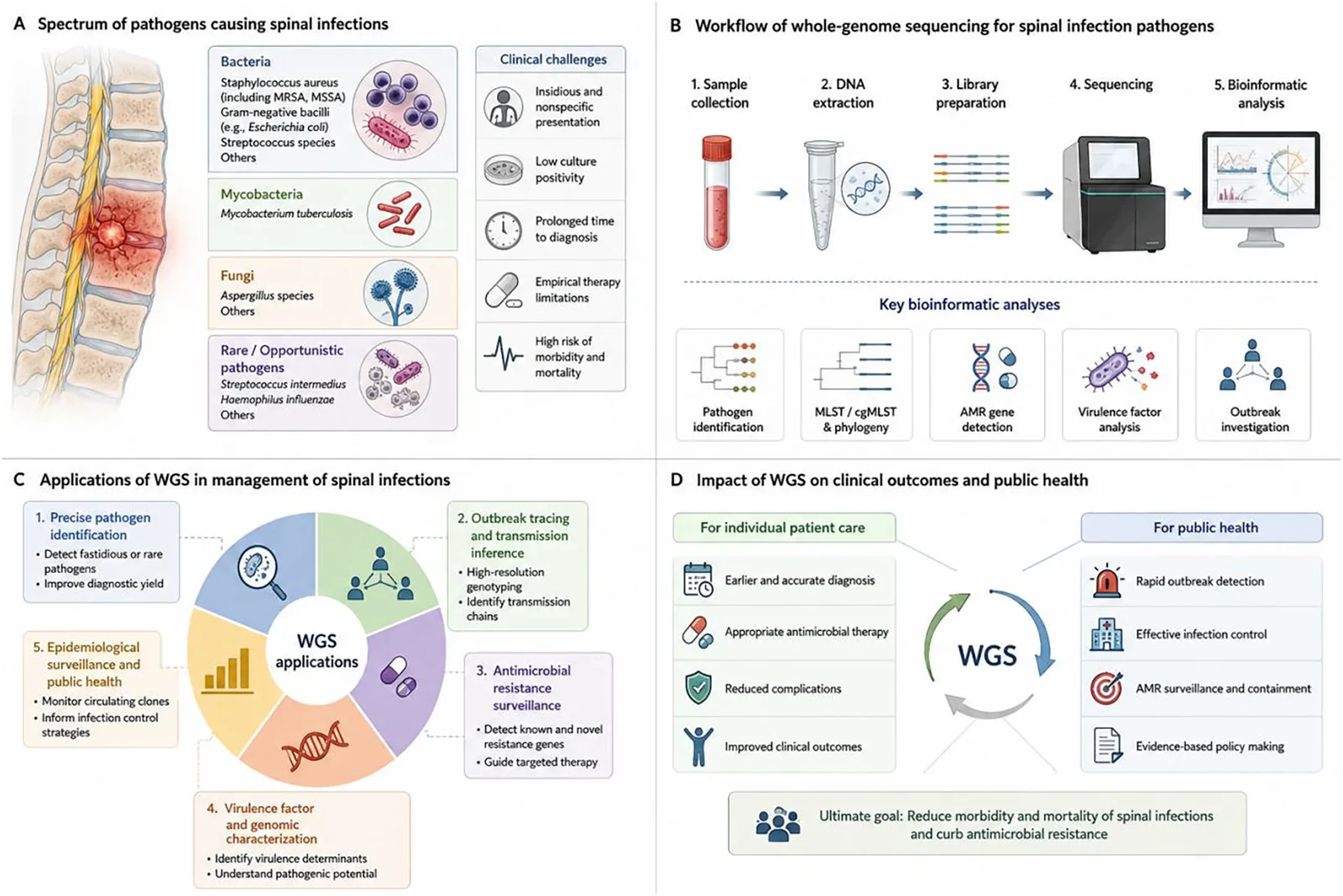
Integrated WGS framework linking pathogen characterization, sequencing workflow, genomic epidemiology, and clinical and public-health applications. (**A**: Spectrum of pathogens causing spinal infections; **B**: Workflow of whole-genome sequencing for spinal infection pathogens; **C**: Applications of WGS in management of spinal infections; **D**: Impact of WGS on clinical outcomes and public health).

## 3 Application of whole genome sequencing in outbreak tracing of spinal infections

### 3.1 The central role of single nucleotide polymorphism analysis in inferring transmission chains

Whole-genome sequencing (WGS) offers unparalleled resolution at the single-nucleotide level, enabling precise differentiation among bacterial strains by analyzing core genome single nucleotide polymorphisms (cgSNPs). This high-resolution genotyping facilitates quantification of genetic distances between isolates, which is critical for reconstructing transmission relationships and directions in outbreak investigations. In the context of spinal infections, where pathogens often exhibit subtle genetic variations, cgSNP analysis serves as a robust tool to discern recent common ancestry and ongoing transmission events. Empirically, when isolates from different patients differ by fewer than a defined SNP threshold—commonly 10 to 15 SNPs—this strongly suggests a recent shared source and active spread within a population. It should be highlighted that 10-15 SNP is an empirical cutoff for common bacterial species. This threshold cannot be mechanically applied to all pathogens; optimal SNP cut-offs vary by bacterial species, sample type, and sequencing quality. This SNP cutoff has become a widely accepted standard in genomic epidemiology for outbreak delineation. Compared to traditional typing methods such as pulsed-field gel electrophoresis (PFGE), which may yield false-positive clustering due to limited discriminatory power, cgSNP analysis enhances specificity and accuracy by effectively distinguishing epidemiologically linked strains from unrelated sporadic cases. For example, in studies of osteomyelitis and spondylitis caused by pathogens such as Staphylococcus aureus and Brucella melitensis, cgSNP-based phylogenetic analyses have successfully identified transmission clusters and ruled out coincidental cases, thereby refining outbreak detection and guiding targeted infection control measures (Walker et al., 2021; Peng et al., 2024; Zhu et al., 2024). This precision is particularly valuable in spinal infections where clinical and microbiological presentations may overlap, and where timely identification of transmission chains can prevent further spread and improve patient outcomes. Thus, cgSNP analysis via WGS stands as a cornerstone in the genomic tracing of spinal infection outbreaks, enabling detailed reconstruction of pathogen dissemination with single-nucleotide resolution.

### 3.2 Practical value of phylogenetic analysis in tracing cross-regional transmission

Phylogenetic analysis based on whole genome sequences provides a powerful framework to visualize and interpret the evolutionary relationships among bacterial isolates from diverse geographic and temporal origins. Construction of maximum likelihood or Bayesian phylogenetic trees enables researchers to map the genetic relatedness and infer the spatiotemporal dynamics of pathogen spread. In spinal infection outbreaks, such analyses can reveal how specific clones disseminate across regions or countries, offering critical insights into cross-border transmission pathways. By integrating phylogenetic data with Bayesian ancestral state reconstruction, it becomes possible to estimate the timing and geographic origin of the most recent common ancestor of outbreak strains, thereby reconstructing the spatiotemporal trajectory of pathogen expansion. This approach elucidates clonal expansion patterns and transmission routes that are otherwise difficult to discern through conventional epidemiological methods. A compelling example is the case of a Japanese traveler who developed purulent spondylitis and bilateral psoas abscesses caused by Streptococcus suis sequence type (ST) 283. Whole-genome phylogenetic analysis grouped this isolate with strains from Vietnam collected during 2015–2016, precisely tracing the infection source to local acquisition in Vietnam. This case underscores the utility of WGS-based phylogenetics in constructing cross-regional transmission chains and highlights its role in identifying zoonotic spillover events and tracking international dissemination of pathogens (He et al., 2026). Such phylogenetic frameworks are invaluable for public health surveillance, enabling early detection of emerging clones with epidemic potential and informing coordinated control strategies across jurisdictions. Therefore, phylogenetic analysis grounded in WGS data is indispensable for understanding and managing the complex transmission dynamics of spinal infection pathogens on a global scale.

### 3.3 Application models and case studies of genomic epidemiology in outbreak investigations

Genomic epidemiology integrates pathogen whole-genome sequencing data with detailed epidemiological information—such as timing, location, exposure history, and contact networks—to enhance outbreak investigations. This integrative approach markedly improves the efficiency and precision of tracing transmission events, shifting outbreak response from reactive to proactive control. In healthcare-associated spinal infection outbreaks, WGS enables discrimination between nosocomial and community-acquired strains, identification of transmission vectors, and detection of persistent environmental reservoirs. For instance, in hospital outbreaks involving methicillin-resistant Staphylococcus aureus (MRSA) or carbapenemase-producing Klebsiella pneumoniae, WGS has been instrumental in pinpointing clonal clusters and guiding targeted interventions to interrupt transmission chains (Sattler et al., 2024). Moreover, large-scale comparative analyses leveraging public genome databases, such as the NCBI Pathogen Detection system, facilitate contextualization of local outbreak strains within global clonal lineages, thereby assessing international public health implications and prioritizing control efforts. Despite these advances, current applications of WGS in outbreak tracing predominantly rely on retrospective analyses. The advent and increasing accessibility of real-time sequencing technologies promise to revolutionize outbreak management by enabling prospective surveillance and immediate tracking of pathogen spread. This evolution will foster dynamic outbreak response models, particularly for spinal infections where rapid identification of transmission sources is critical to prevent severe complications. Collectively, genomic epidemiology, through the integration of WGS and epidemiological data, offers a transformative paradigm for outbreak investigation and control, with demonstrated success in spinal infection contexts and promising future applications as sequencing technologies become more rapid and accessible (Zhu et al., 2024; Wu et al., 2025).

## 4 Application of whole genome sequencing in monitoring antimicrobial resistance transmission in spinal infections

### 4.1 Precise genomic analysis of resistance genes and correlation with resistance phenotypes

Whole-genome sequencing (WGS) offers a comprehensive approach to simultaneously scan all antimicrobial resistance (AMR) genes present in pathogens responsible for spinal infections. This panoramic analysis includes acquired resistance genes, chromosomal mutations conferring resistance, and genes related to efflux pump systems, enabling a full-spectrum understanding of the resistome. For example, in Enterococcus cecorum isolates from(animal-derived isolates) broilers with osteomyelitis, WGS revealed resistance determinants linked to vancomycin, tetracycline, lincomycin, spectinomycin, and tylosin, many of which were located on mobile genetic elements (MGEs) such as integrative conjugative elements and genomic islands, highlighting the complex genomic context of resistance genes (Huang et al., 2024). Similarly, coagulase-negative staphylococci (CoNS)(animal-derived isolates) from bone lesions in broiler chickens exhibited diverse AMR genes including blaZ, tet(L), tet(M), and erm(C), with WGS uncovering their association with mobile elements and providing insights into phenotypic resistance profiles (Szafraniec et al., 2026).

By integrating WGS data with phenotypic antimicrobial susceptibility testing, systematic genotype-phenotype correlation models can be constructed. These models enhance the predictive accuracy for resistance phenotypes based on detected genetic determinants, thereby guiding clinicians in selecting effective antibiotic regimens for spinal infections. For instance, in methicillin-resistant Staphylococcus aureus (MRSA) isolates from osteomyelitis cases, WGS identified mutations and polymorphisms in resistance and virulence genes that correlated with multidrug resistance and enhanced pathogenicity, which was consistent with phenotypic resistance profiles (Peng et al., 2024). Moreover, WGS enables quantification of resistance gene expression regulation and assessment of their genetic environment. Analysis of upstream insertion sequences, promoter mutations, and gene copy number variations elucidates molecular mechanisms underlying phenotypic variability in resistance. For example, amplification and mutation of blaKPC genes in carbapenem-resistant Klebsiella pneumoniae during prolonged infection were linked to increased ceftazidime/avibactam resistance and competitive fitness advantages, as revealed by WGS (Yang et al., 2025).

Furthermore, precise gene annotation through WGS allows identification of subtypes of resistance gene clusters and their functional characteristics. Detailed genomic localization of β-lactamase genes in Mangrovibacter phragmitis explained its resistance to ampicillin and cefotaxime, providing critical information for clinical decision-making (Cummins et al., 2023). Collectively, WGS facilitates a panoramic and precise characterization of resistance determinants and their phenotypic manifestations in spinal infection pathogens, enabling personalized antimicrobial therapy and improved clinical outcomes.

### 4.2 Molecular mechanisms of horizontal transfer of resistance genes and risk assessment of dissemination

WGS is instrumental in systematically identifying mobile genetic elements (MGEs) such as plasmids, transposons, insertion sequences, and integrons within pathogen genomes, which mediate horizontal gene transfer (HGT) of resistance genes. This capability provides direct molecular evidence elucidating the mechanisms by which resistance determinants disseminate among bacterial strains causing spinal infections. For example, in Enterococcus cecorum isolates (animal-derived isolates) associated with broiler osteomyelitis, WGS revealed that multidrug-resistant clones clustered phylogenetically and that integrative conjugative elements and genomic islands were the primary carriers of AMR genes, underscoring the role of MGEs in resistance spread (Laurentie et al., 2023).

Integrated genomic analyses enable mapping of co-localization between resistance genes and MGEs, including characterization of flanking sequences, which is critical for assessing the transfer potential and epidemiological risk of resistance dissemination. For instance, in methicillin-resistant Staphylococcus aureus (MRSA) ST45 isolates from osteomyelitis, WGS detected transposable elements IS256 and IS257R2 uniquely in osteomyelitis-associated strains, suggesting their involvement in resistance gene mobility and pathogenicity (Peng et al., 2024). Comparative genomics further allows tracking of specific resistance genes across different strains, species, and ecological niches, revealing transmission chains that span environmental, animal, and human interfaces. This approach supports the One Health framework by providing data to inform integrated antimicrobial resistance control strategies.

A notable example includes the identification of the tetM resistance gene located on transposon-related elements in Streptococcus agalactiae ST283 strains, indicating its potential for tetracycline resistance dissemination via horizontal transfer (Szafraniec et al., 2026). Such molecular evidence is crucial for evaluating the risk of resistance gene spread within spinal infection patient populations and beyond. By leveraging WGS to dissect the molecular architecture and mobility of resistance determinants, surveillance systems can more accurately predict and mitigate the propagation of antimicrobial resistance in clinical and community settings.

### 4.3 International surveillance and early warning of resistant clonal lineages

The integration of WGS with population genomics enables precise identification and characterization of high-risk resistant clonal lineages implicated in spinal infections, elucidating their global distribution and evolutionary dynamics. This genomic epidemiology approach supports international collaborative surveillance efforts by providing high-resolution data on the dissemination patterns of resistant clones. For example, global phylogenetic analyses of carbapenemase-producing Klebsiella pneumoniae isolates revealed the presence of hybrid plasmids harboring both resistance and virulence genes, with certain lineages causing outbreaks, including cases of osteomyelitis, emphasizing the need for coordinated monitoring (Sattler et al., 2024).

Constructing global phylogenomic frameworks based on WGS data facilitates rapid classification and risk assessment of emerging resistant strains. This capability enables early warning systems to detect novel resistance variants and prioritize resource allocation for infection control. Continuous WGS surveillance allows dynamic tracking of clonal spread across regions, assessment of evolutionary rates, and evaluation of intervention effectiveness. For instance, WGS-based monitoring of Salmonella enterica ST376 isolates from disseminated infections demonstrated within-host diversification and regional transmission patterns, informing public health strategies (Saleem et al., 2026).

Despite these advances, there remains a lack of dedicated global genomic surveillance networks specifically targeting resistant clones causing spinal infections. Establishing specialized genomic databases and standardized analytical pipelines tailored to orthopedic infections would enhance the sensitivity and timeliness of resistance monitoring and outbreak detection. Such infrastructure would improve pathogen source tracing and support international efforts to curb antimicrobial resistance in spinal infections. Overall, WGS-driven international surveillance and early warning systems represent critical tools for managing the global threat of resistant clonal lineages in spinal infection contexts (**Figure 3**).

**Figure 3:**
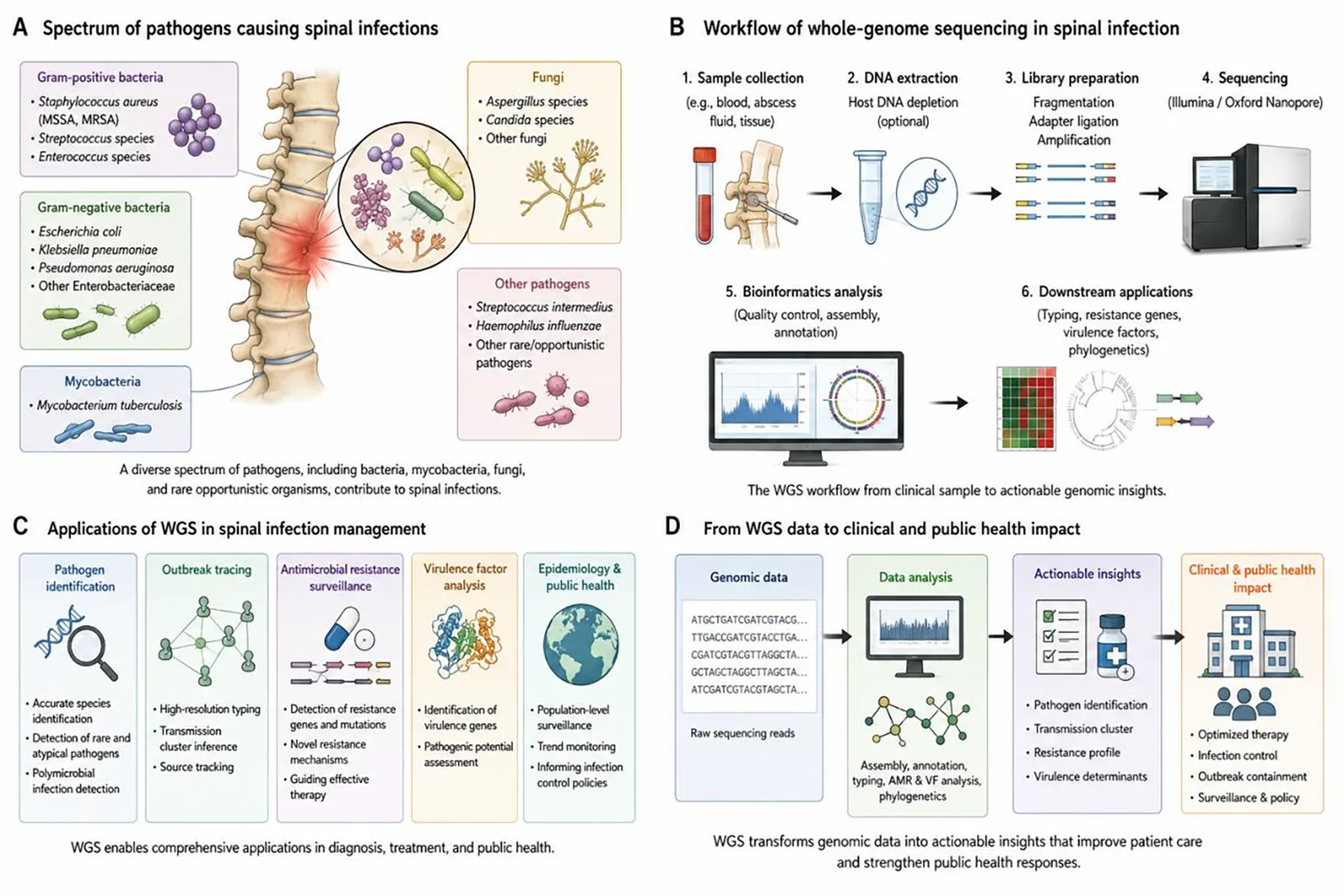
Integrated use of WGS for pathogen identification, outbreak tracing, antimicrobial resistance surveillance, virulence analysis, and clinical translation. (**A**: Spectrum of pathogens causing spinal infections; **B**: Workflow of whole-genome sequencing in spinal infection; **C**: Applications of WGS in spinal infection management; **D**: From WGS data to clinical and public health impact).

## 5 Unique value of whole genome sequencing in identification of rare and emerging pathogens

### 5.1 Genomic identification of rare pathogens in spinal infections

Rare pathogens causing spinal infections present significant diagnostic challenges due to their low prevalence and the limitations of conventional phenotypic identification methods. Traditional approaches, such as culture-based techniques and targeted immunological assays, often rely on pre-established probes or phenotypic markers, which may be insufficient or misleading when encountering uncommon or novel organisms. Whole-genome sequencing (WGS), by contrast, offers an unbiased, probe-independent platform capable of precise species-level identification and phylogenetic placement of any pathogen present in clinical specimens. This capability is particularly critical in spinal infections where rare pathogens may be overlooked or misclassified, leading to inappropriate treatment and poor outcomes.

A notable example is the identification of Pythium insidiosum in rare human spinal infections. Conventional morphological and immunological methods have struggled to accurately detect this pathogen due to its atypical characteristics and resemblance to fungal organisms. WGS overcomes these limitations by enabling comprehensive genomic analysis, which not only confirms the presence of P. insidiosum but also allows its precise phylogenetic positioning alongside closely related strains such as MCC 13. This genomic insight provides a technical paradigm for the accurate diagnosis of infections caused by fungal-like pathogens, facilitating targeted therapeutic strategies and improving clinical management.

Moreover, WGS combined with molecular metrics such as average nucleotide identity (ANI) analysis enhances the discrimination of closely related species that exhibit overlapping phenotypic and biochemical profiles. For instance, the differentiation of Mangrovibacter phragmitis from the Enterobacter cloacae complex, a group notoriously difficult to resolve by traditional methods, has been successfully achieved through WGS and ANI. This precise taxonomic resolution is essential for understanding the genetic basis of pathogenicity and antimicrobial resistance, as well as for elucidating the clinical relevance of newly emerging pathogens.

Compared to conventional Sanger sequencing, which typically targets single gene loci such as 16S rRNA, WGS leverages the entire genome sequence, offering superior resolution and comprehensive information for species delineation and evolutionary studies. This holistic genomic approach significantly improves the accuracy of rare pathogen identification in spinal infections, thereby enhancing clinical decision-making and patient outcomes. The integration of WGS into diagnostic workflows addresses the critical gap left by traditional methods, enabling the detection of rare and emerging pathogens that would otherwise remain undiagnosed or misclassified (Nienhold et al., 2022; Purushothaman et al., 2022).

### 5.2 Genomic analysis of virulence factors and pathogenic mechanisms

Whole-genome sequencing provides a panoramic view of the virulence repertoire encoded within pathogen genomes, encompassing adhesins, invasins, toxins, secretion systems, and immune evasion factors. This comprehensive genomic profiling is instrumental in elucidating the molecular underpinnings of pathogenicity in spinal infections, where understanding the interplay of virulence determinants is key to developing effective interventions.

For example, genomic analysis of Mangrovibacter phragmitis PSU-3885-11 revealed the presence of critical virulence genes such as ompA, hcp/tssD, and rpoS. The ompA gene encodes an outer membrane protein implicated in bacterial adherence and biofilm formation, facilitating colonization of spinal tissues. The hcp/tssD gene is part of the type VI secretion system, which mediates interbacterial interactions and host cell manipulation, potentially contributing to immune evasion and persistence. The rpoS gene encodes a sigma factor involved in stress response regulation, enhancing bacterial survival under hostile conditions within the host. The identification of these genes underscores their probable roles in colonization, immune escape, and chronic infection in spinal disease contexts.

Pangenomic analyses further enable comparative studies across strains from diverse sources, revealing patterns of virulence gene acquisition or loss that distinguish clinical isolates from environmental or commensal counterparts. Such differential virulence gene clusters may serve as biomarkers for pathogenic potential and inform risk stratification in spinal infections. This approach also aids in identifying novel therapeutic targets by pinpointing virulence determinants uniquely associated with clinical strains.

Advancements in artificial intelligence, particularly the integration of protein language models with deep learning classifiers, have introduced innovative methodologies for predicting and quantifying the virulence potential of rare pathogens based on genomic data. These AI-driven tools complement experimental assays by providing rapid, high-throughput functional predictions, thereby accelerating the characterization of pathogenic mechanisms and guiding experimental validation efforts.

Collectively, WGS-based virulence profiling offers a gene-level panoramic insight into pathogen biology, facilitating a deeper understanding of the mechanisms driving spinal infections and supporting the development of precision therapeutics and preventive strategies (Guitart-Matas et al., 2022; Purushothaman et al., 2022).

### 5.3 Construction of genomic resource repositories for emerging spinal infection pathogens

The generation and open sharing of whole-genome sequences from rare spinal infection pathogens constitute invaluable public health resources that catalyze global research and clinical advancements. Each sequenced isolate enriches the collective genomic repository, enabling enhanced recognition, diagnosis, and surveillance of these uncommon agents worldwide.

For instance, the genome of Weissella viridescens Wvir453, representing the third globally reported clinical isolate and the first with a publicly available genome, provides critical reference data. This genomic resource facilitates assessment of the species’ zoonotic potential and informs safety evaluations of related probiotic products, underscoring the translational impact of such repositories.

Effective genomic resource construction necessitates systematic integration of sequence data, comprehensive metadata, and standardized analytical outputs. Adherence to FAIR (Findable, Accessible, Interoperable, and Reusable) data management principles ensures that genomic datasets are discoverable and usable by diverse stakeholders, fostering collaborative research and rapid knowledge dissemination.

Moreover, multidimensional integration of pathogen genomic data with clinical phenotypes, therapeutic outcomes, and epidemiological information enables the development of knowledge-driven precision control frameworks. Such integrative platforms maximize the clinical and public health utility of genomic data, supporting tailored diagnostic algorithms, outbreak investigations, and antimicrobial stewardship in spinal infection management.

In summary, the establishment of robust, accessible genomic databases for rare and emerging spinal infection pathogens is foundational to advancing diagnostic accuracy, understanding pathogen biology, and enhancing global surveillance and control efforts (Purushothaman et al., 2022; Spruijtenburg et al., 2022).

## 6 Challenges and prospects of whole genome sequencing applications

### 6.1 Bottlenecks in technical standardization and bioinformatics analysis

Whole-genome sequencing (WGS) technology currently faces significant challenges related to the lack of unified operational protocols and quality control standards, which critically impact its clinical application in spinal infection diagnostics. Variability in DNA extraction methods, library preparation techniques, sequencing platforms, and sequencing depth across different laboratories leads to inconsistencies in data comparability and reproducibility. This heterogeneity restricts the widespread adoption of WGS as a routine clinical diagnostic tool for spinal infections. For instance, proficiency testing programs have revealed diverse bioinformatics pipelines and variable data quality among clinical and public health laboratories, underscoring the urgent need for standardized workflows and benchmarking standards to ensure reliable pathogen identification and antimicrobial resistance (AMR) detection (Lau et al., 2021). The complexity of bioinformatics analysis remains a major bottleneck in clinical translation. Currently, there is a paucity of standardized, validated, and user-friendly bioinformatics tools that can seamlessly integrate pathogen identification, resistance gene detection, and single nucleotide polymorphism (SNP) analysis. Different software and parameter settings often yield divergent results, complicating the interpretation of resistance genotypes and outbreak investigations. Moreover, the storage, management, and sharing of large-scale sequencing data pose infrastructural and ethical challenges. Healthcare institutions must develop secure, efficient data management systems that comply with privacy regulations while facilitating clinical needs and inter-institutional data sharing. Additionally, the unique microbial diversity associated with spinal infections necessitates the establishment of specialized reference genome databases and lineage typing systems to enhance the accuracy and adaptability of genomic analyses. Such tailored genomic resources would improve the resolution of pathogen diversity and evolutionary relationships, thereby strengthening outbreak source tracing and resistance monitoring (On and Yoon, 2026). Collectively, addressing these technical and bioinformatics bottlenecks through standardization, infrastructure development, and specialized database creation is essential to unlock the full potential of WGS in spinal infection management.

### 6.2 Balancing clinical accessibility and cost-effectiveness

Despite the progressive reduction in sequencing costs, the clinical implementation of WGS in microbiology laboratories, particularly for spinal infections, remains constrained by substantial investments in equipment, scarcity of specialized personnel, and high operational and maintenance expenses. The sequencing turnaround time, encompassing both data generation and analysis, typically spans several days, which conflicts with the urgent therapeutic windows characteristic of acute spinal infections requiring rapid antimicrobial intervention. This temporal limitation diminishes the immediate clinical utility of WGS in guiding timely treatment decisions. Economic evaluations underscore the necessity for comprehensive clinical validation and health economic assessments to justify the integration of WGS into routine diagnostics. Notably, dedicated health-economic evidence specifically for WGS implementation in human spinal infection cohorts remains scarce; future prospective real-world studies are required to quantify cost-benefit in this patient population. Prospective studies are needed to quantify the impact of WGS on patient outcomes, treatment optimization, and overall healthcare expenditure in spinal infection cohorts (Price et al., 2023). Technological advancements such as platform integration and automation of sequencing workflows hold promise for reducing turnaround times and costs. The maturation of real-time sequencing technologies, including nanopore-based platforms, offers the potential for bedside or point-of-care applications, enhancing accessibility in primary and resource-limited healthcare settings (Wu et al., 2025). These innovations could reconcile the demand for rapid, cost-effective diagnostics with the complexity of WGS, facilitating its broader clinical adoption. Ultimately, strategic investments in infrastructure, workforce training, and technology development are critical to achieving a sustainable balance between clinical accessibility and cost-effectiveness of WGS in spinal infection diagnostics.

### 6.3 Uncertainties in correlating resistance genotypes with clinical phenotypes

A significant challenge in applying WGS for antimicrobial resistance surveillance in spinal infections lies in the incomplete understanding of the expression regulation and functional contributions of many resistance genes. Newly identified resistance determinants or mutations often lack corresponding phenotypic validation, resulting in discrepancies between genotypic predictions and actual antimicrobial susceptibility testing outcomes. This discordance complicates clinical decision-making based on genomic data alone. The phenomenon of heterogeneous resistance and the influence of *in vivo* microenvironments further confound genotype-phenotype correlations. Within spinal infection foci, biofilm formation on vertebral bone and implanted spinal devices can shield pathogens from antibiotic exposure. This local microenvironment may lead to clinical treatment failure even when genomic prediction suggests susceptibility, complicating genotype-phenotype correlation. For example, antibiotics that appear effective *in vitro* may fail clinically due to factors such as biofilm formation, acidic pH, or immune evasion mechanisms within infected spinal tissues (On and Yoon, 2026). In rare pathogens implicated in spinal infections, such as Pythium insidiosum, WGS-predicted resistance profiles are often unsupported by robust pharmacological data, and the translation of minimum inhibitory concentration (MIC) values into effective clinical dosing remains uncertain. To overcome these limitations, the accumulation of comprehensive prospective datasets that integrate complete clinical information, antimicrobial susceptibility profiles, and treatment outcomes is imperative. The application of machine learning and multi-omics integration can facilitate the development of more precise predictive models of resistance phenotypes, enabling a nuanced understanding of complex resistance mechanisms and improving the reliability of WGS-based resistance predictions (Purushothaman et al., 2022). Such advances will enhance the clinical utility of WGS in guiding targeted antimicrobial therapy for spinal infections.

### 6.4 Multi-omics integration and technological innovations

The integration of WGS with complementary omics technologies—such as transcriptomics, proteomics, and metabolomics—enables the construction of multilayered “genotype-phenotype-function” networks that provide comprehensive insights into the pathogenicity and resistance evolution of spinal infection microorganisms. This holistic approach facilitates the elucidation of molecular mechanisms underlying infection and antimicrobial resistance, surpassing the resolution achievable by genomics alone (Purushothaman et al., 2022). Metagenomic sequencing, which captures the entire microbial community directly from clinical specimens without the need for culture, holds particular promise for spinal infections where pathogens may be fastidious or patients have received prior antibiotic therapy. This culture-independent method can reveal the composition and functional potential of pathogen populations, enhancing diagnostic accuracy and outbreak investigation (Lau et al., 2021). Third-generation long-read sequencing technologies, such as PacBio and Oxford Nanopore, offer the advantage of generating contiguous sequences spanning repetitive regions and complex genomic structures, including plasmids and genomic islands harboring resistance genes. This capability improves the assembly and characterization of mobile genetic elements, facilitating precise detection of horizontal gene transfer events that contribute to resistance dissemination (Goraichuk et al., 2024). Furthermore, artificial intelligence, particularly deep learning algorithms, is increasingly applied to genome annotation, resistance phenotype prediction, and outbreak cluster discrimination. These AI-driven tools enable cross-species and cross-platform analyses, delivering intelligent, visualized interpretations of WGS data that significantly enhance the effectiveness of pathogen surveillance and control in spinal infections (On and Yoon, 2026). Collectively, the fusion of multi-omics data and cutting-edge technologies heralds a new era of precision diagnostics and epidemiological insights in spinal infection management.

### 6.5 Clinical translation and construction of precision prevention systems

Integrating WGS into the diagnostic and therapeutic decision-making pathways for spinal infections enables a unified solution encompassing pathogen identification, resistance profiling, virulence assessment, and outbreak source tracing. This integration substantially shortens the time required to formulate precise treatment strategies, thereby improving patient outcomes. Establishing a closed-loop management system encompassing sequencing, analysis, reporting, and clinical decision-making is vital. Developing multidisciplinary teams proficient in microbiology, genomics, and clinical medicine is essential to facilitate the transition of WGS from a research tool to a routine clinical diagnostic modality (Ferdinand et al., 2021). Prospective, multicenter, and multidisciplinary cohort studies focusing on the genomic epidemiology of spinal infections are necessary to build shared databases that combine clinical outcomes with genomic data. Such resources will support evidence-based refinement of prevention and control strategies, expanding WGS surveillance from individual hospitals to regional and national networks (Ferdinand et al., 2021). Meanwhile, multi-center genomic-clinical databases must strictly comply with patient privacy regulations. Secure de-identification workflows are essential before data sharing to mitigate ethical risks mentioned in the previous section. Ultimately, constructing a precision prevention system centered on WGS will transform spinal infection management from reactive to proactive, shifting from empirical treatment to targeted therapy and from individual patient care to population-level control. This paradigm shift promises to reduce the burden of spinal infections and curb the spread of antimicrobial resistance, marking a significant advancement in infectious disease control (**Figure 4**).

**Figure 4:**
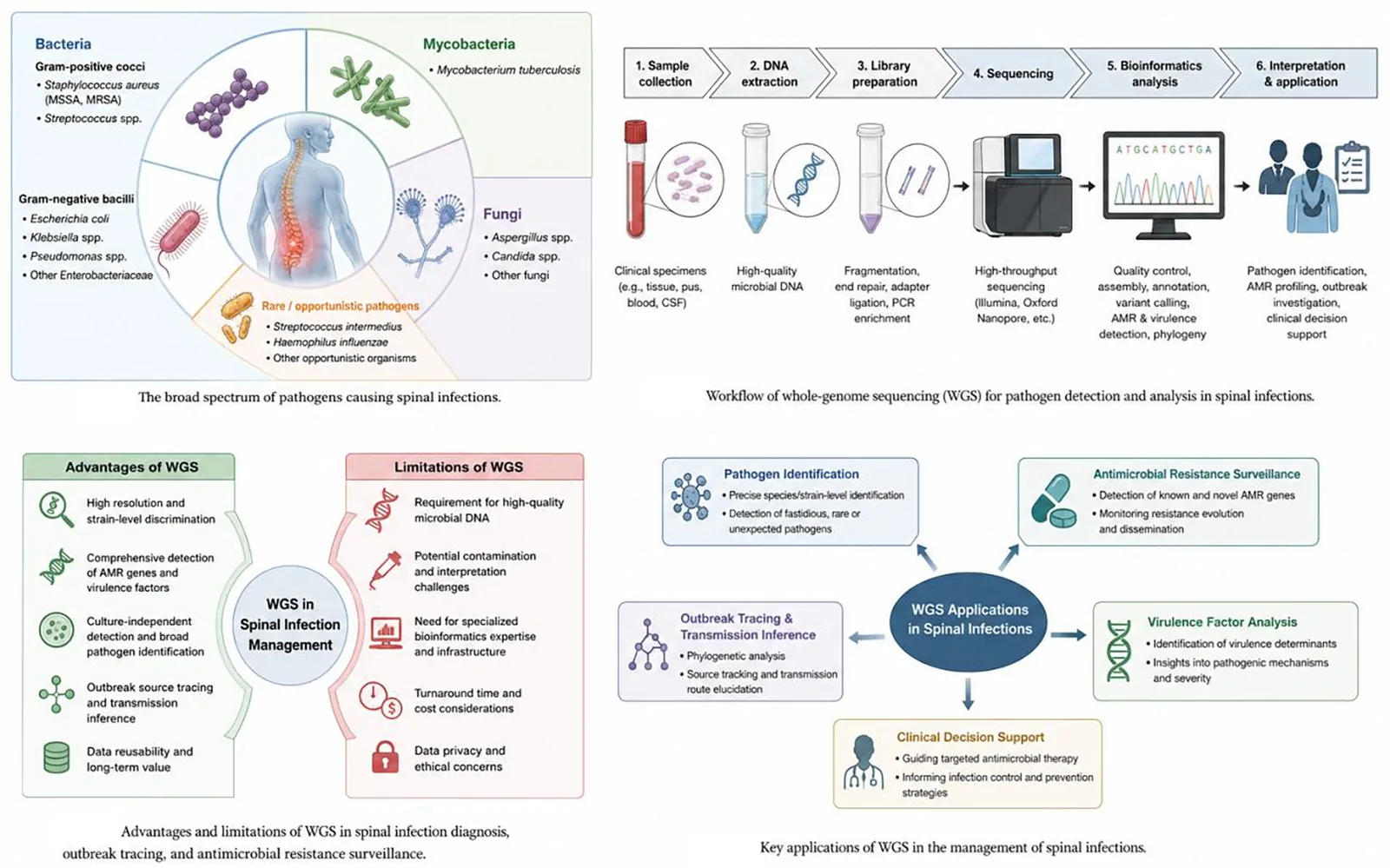
Summary of pathogen spectrum, WGS workflow, advantages and limitations, and key applications in spinal infections.

## 7 Conclusion

The advent of whole-genome sequencing (WGS) has revolutionized the field of spinal infection research and clinical management by providing unprecedented resolution and comprehensive genomic insights into the causative pathogens. From an expert perspective, the integration of WGS into the study of spinal infections marks a paradigm shift, enabling precise pathogen identification at single-nucleotide resolution, robust phylogenetic tracing, and in-depth elucidation of antimicrobial resistance (AMR) mechanisms. This technological advancement has not only enhanced our understanding of pathogen biology but also transformed molecular epidemiology approaches, offering powerful tools for outbreak investigation and resistance surveillance.

Balancing the diverse research perspectives, it is evident that WGS excels in several critical domains. Firstly, its application in outbreak source tracing has demonstrated remarkable capability in reconstructing transmission chains and mapping cross-regional dissemination pathways. This molecular epidemiological strength facilitates rapid recognition of spinal infection outbreaks and informs targeted intervention strategies, thereby improving patient outcomes and public health responses. Secondly, WGS provides a panoramic view of resistance gene landscapes, uncovering the molecular underpinnings of horizontal gene transfer events that drive AMR spread. Such insights are invaluable for developing evidence-based antimicrobial stewardship policies and tailoring therapeutic regimens to combat resistant infections effectively.

Moreover, WGS has proven indispensable in identifying rare and emerging spinal infection pathogens, surpassing traditional diagnostic methods that often lack sensitivity or specificity. The establishment of comprehensive genomic repositories through WGS data accumulation lays a foundational framework for future research and surveillance, enabling early detection and containment of novel infectious threats. This aspect underscores the transformative potential of WGS in expanding the diagnostic horizon and enhancing preparedness against evolving pathogen profiles.

Despite these significant advances, the translation of WGS from research settings to routine clinical practice in spinal infections faces several challenges. Technical standardization remains a critical hurdle, as variability in sequencing platforms, data processing pipelines, and interpretation criteria can affect reproducibility and comparability across studies and institutions. Additionally, cost-effectiveness considerations continue to influence the feasibility of widespread clinical adoption, particularly in resource-limited settings. The complex relationship between genotype and phenotype also poses interpretative challenges; correlating genomic data with clinical manifestations and treatment responses requires further elucidation through integrative multi-omics and longitudinal studies.

Looking forward, the trajectory of WGS in spinal infection management is promising, driven by ongoing reductions in sequencing costs, optimization of analytical workflows, and the integration of complementary omics technologies. The incorporation of artificial intelligence and machine learning algorithms holds particular promise for enhancing data interpretation, enabling rapid, accurate, and clinically actionable insights. To fully realize the clinical potential of WGS, concerted efforts must focus on establishing multicenter collaborative networks and standardized protocols. Building comprehensive spinal infection pathogen genomic databases and high-throughput surveillance platforms will facilitate real-time monitoring of outbreaks and resistance trends, fostering a proactive rather than reactive approach to infection control. It should be objectively recognized that existing WGS applications for spinal infections are largely derived from retrospective investigations. Large-scale multi-center prospective cohort evidence confirming the clinical prognostic benefits of WGS remains limited and warrants further research.

In conclusion, whole-genome sequencing stands at the forefront of precision medicine in spinal infections, offering unparalleled capabilities for pathogen characterization, outbreak investigation, and resistance monitoring. While challenges remain in standardization, cost, and genotype-phenotype integration, the synergistic advancement of sequencing technologies, bioinformatics, and collaborative infrastructures is poised to overcome these barriers. The future of spinal infection management lies in harnessing WGS as a routine clinical tool, enabling precise, timely, and effective interventions that ultimately improve patient care and public health outcomes. This balanced integration of cutting-edge genomic science with clinical practice will usher in a new era of precision infectious disease control, transforming the landscape of spinal infection diagnosis, treatment, and prevention.

## Data Availability

The original contributions presented in the study are included in the article/supplementary material, further inquiries can be directed to the corresponding author/s.

## References

[B1] Araujo A. C. Cho A. Y. Silva L. M. N. Corrêa T. C. Souza G. C. Albuquerque A. S. . (2024). Mortality in sea lions is associated with the introduction of the H5N1 clade 2.3.4.4b virus in Brazil october 2023: Whole genome sequencing and phylogenetic analysis. BMC Vet. Res. 20, 285. doi: 10.1186/s12917-024-04137-1 38956597 PMC11221036

[B2] Cummins M. L. Li D. Ahmad A. Bushell R. Noormohammadi A. H. Wijesurendra D. S. . (2023). Whole genome sequencing of avian pathogenic escherichia coli causing bacterial chondronecrosis and osteomyelitis in Australian poultry. Microorganisms 11. doi: 10.3390/microorganisms11061513 37375015 PMC10302171

[B3] Douan J. Kohler C. Haralambiev L. Idelevich E. A. Becker K. (2026). Comparative phylogenetic, antimicrobial resistance, and clinical characterization of human spondylodiscitis-associated staphylococcus pseudintermedius. Front. Microbiol. 17, 1735075. doi: 10.3389/fmicb.2026.1735075 41783499 PMC12953532

[B4] Fayad S. Daaboul D. Kassem I. I. Abbara A. Cazer C. L. Fiorella K. J. . (2025). Evidence of transmission and dissemination of diverse bla NDM-5 -producing escherichia coli clones between refugee and host communities and their environment: A multicenter cross-sectional study. Appl. Environ. Microbiol. 91, e0162525. doi: 10.1128/aem.01625-25 41186404 PMC12724354

[B5] Ferdinand A. S. Kelaher M. Lane C. R. Silva A. G. Sherry N. L. Ballard S. A. . (2021). An implementation science approach to evaluating pathogen whole genome sequencing in public health. Genome Med. 13, 121. doi: 10.1186/s13073-021-00934-7 34321076 PMC8317677

[B6] Goraichuk I. V. Risalvato J. Pantin-Jackwood M. Suarez D. L. (2024). Improved influenza a whole-genome sequencing protocol. Front. Cell. Infect. Microbiol. 14, 1497278. doi: 10.3389/fcimb.2024.1497278 39669272 PMC11635996

[B7] Guitart-Matas J. Gonzalez-Escalona N. Maguire M. Vilaró A. Martinez-Urtaza J. Fraile L. . (2022). Revealing genomic insights of the unexplored porcine pathogen actinobacillus pleuropneumoniae using whole genome sequencing. Microbiol. Spectr. 10, e0118522. doi: 10.1128/spectrum.01185-22 35856711 PMC9430968

[B8] He P. Wang H. Li P. Yan Y. Gao L. Chen L. (2026). Pyogenic spondylitis with epidural abscess caused by streptococcus suis serotype 2 ST7: Tissue mNGS confirmation and whole-genome characterization of a human isolate. Pathog. (Basel Switzerland) 15. doi: 10.3390/pathogens15030314 41901767 PMC13028986

[B9] Huang Y. Boyen F. Antonissen G. Vereecke N. Immerseel F. V. (2024). The genetic landscape of antimicrobial resistance genes in enterococcus cecorum broiler isolates. Antibiotics (Basel Switzerland) 13. doi: 10.3390/antibiotics13050409 38786138 PMC11117384

[B10] Ibrahim O. Aboushaala R. Ahmed N. Savoia A. Ward S. O. Patel S. N. . (2026). The diagnostic value of metagenomic next-generation sequencing versus traditional microbiological testing in native pyogenic spinal infections: A systematic review and meta-analysis. North. Am. Spine Soc. J. 25, 100840. doi: 10.1016/j.xnsj.2025.100840 41584315 PMC12830174

[B11] Lau K. A. Silva A. G. Theis T. Gray J. Ballard S. A. Rawlinson W. D. (2021). Proficiency testing for bacterial whole genome sequencing in assuring the quality of microbiology diagnostics in clinical and public health laboratories. Pathology 53, 902–911. doi: 10.1016/j.pathol.2021.03.012 34274166

[B12] Laurentie J. Loux V. Hennequet-Antier C. Chambellon E. Deschamps J. Trotereau A. . (2023). Comparative genome analysis of enterococcus cecorum reveals intercontinental spread of a lineage of clinical poultry isolates. mSphere 8, e0049522. doi: 10.1128/msphere.00495-22 36794931 PMC10117131

[B13] Le S. Tang J. Zhang J. Fu J. Zhang W. He M. . (2025). Long-segment ventral spinal epidural abscesses caused by mycobacterium tuberculosis: A report of 2 cases and review. JB JS Open Access 10. doi: 10.2106/JBJS.OA.24.00249 40547101 PMC12178294

[B14] Li Z. Yang S. Li C. Zhang L. Xi N. Li D. . (2026). Metagenomic next-generation sequencing for the diagnosis of suspected spinal infections from biopsy samples: A novel biopsy toolkit design and real-life diagnostic value. Eur. Spine Journal: Off. Publ. Eur. Spine Society Eur. Spinal Deformity Society Eur. Section Cervical Spine Res. Soc. 35, 703–711. doi: 10.1007/s00586-025-09686-z 41407999

[B15] Liu Y. Li L. Yang L. Yang Y. An H. Li D. . (2025). Clinical features and treatment strategies of q fever spinal infection: A pooled analysis of 39 cases and narrative review of the literature. Open Forum Infect. Dis. 12, ofaf584. doi: 10.1093/ofid/ofaf584 41058886 PMC12497565

[B16] Nienhold R. Mensah N. Frank A. Graber A. Koike J. Schwab N. . (2022). Unbiased screen for pathogens in human paraffin-embedded tissue samples by whole genome sequencing and metagenomics. Front. Cell. Infect. Microbiol. 12, 968135. doi: 10.3389/fcimb.2022.968135 36204644 PMC9530700

[B17] On Y. Yoon E.-J. (2026). Strategies for integrating whole-genome sequencing into antimicrobial resistance surveillance. BMC Genomics 27. doi: 10.1186/s12864-026-13004-2 42243657 PMC13455497

[B18] Peng K.-T. Chen P.-C. Chen J.-L. Huang T.-Y. Peng Y.-H. Liu J.-F. . (2024). A comparative phenotypic and genomic analysis of methicillin-resistant staphylococcus aureus ST45 isolates from cellulitis and osteomyelitis in Taiwan. J. Infect. Dis. 230, e568–e578. doi: 10.1093/infdis/jiae096 38409272 PMC11420812

[B19] Price V. Ngwira L. G. Lewis J. M. Baker K. S. Peacock S. J. Jauneikaite E. . (2023). A systematic review of economic evaluations of whole-genome sequencing for the surveillance of bacterial pathogens. Microb. Genomics 9. doi: 10.1099/mgen.0.000947 36790430 PMC9997737

[B20] Purushothaman S. Meola M. Egli A. (2022). Combination of whole genome sequencing and metagenomics for microbiological diagnostics. Int. J. Mol. Sci. 23. doi: 10.3390/ijms23179834 36077231 PMC9456280

[B21] Rule R. Mitton B. Govender N. P. Hoffmann D. Said M. (2021). Spinal epidural abscess caused by aspergillus spp masquerading as spinal tuberculosis in a person with HIV. Lancet Infect. Dis. 21, e356–e362. doi: 10.1016/S1473-3099(20)30979-8 34599872

[B22] Saleem A. Ahbabi S. M. A. Shamsi M. S. A. A. Hassani A. A. Ghazawi A. Khalifa H. O. . (2026). Genomic surveillance captures resistance evolution in salmonella enterica ST376 during prolonged disseminated infection in an immunocompromised patient from the United Arab Emirates. J. Infection Public Health 19, 103254. doi: 10.1016/j.jiph.2026.103254 42167111

[B23] Sattler J. Ernst C. M. Zweigner J. Hamprecht A. (2024). High frequency of acquired virulence factors in carbapenemase-producing klebsiella pneumoniae isolates from a large german university hospital 2013-2021. Antimicrob. Agents Chemother. 68, e0060224. doi: 10.1128/aac.00602-24 39365038 PMC11539210

[B24] Sivertsen A. Dyrhovden R. Tellevik M. G. Bruvold T. S. Nybakken E. Skutlaberg D. H. . (2022). Escherichia marmotae-a human pathogen easily misidentified as escherichia coli. Microbiol. Spectr. 10, e0203521. doi: 10.1128/spectrum.02035-21 35380461 PMC9045135

[B25] Spruijtenburg B. Badali H. Abastabar M. Mirhendi H. Khodavaisy S. Sharifisooraki J. . (2022). Confirmation of fifth candida auris clade by whole genome sequencing. Emerging Microbes Infections 11, 2405–2411. doi: 10.1080/22221751.2022.2125349 36154919 PMC9586689

[B26] Szafraniec G. M. Chrobak-Chmiel D. Adamczyk K. Sułecki K. Dolka B. (2026). Phenotypic and genotypic characterization of antimicrobial resistance in coagulase-negative staphylococci from bone lesions in broiler chickens. BMC Vet. Res. 22. doi: 10.1186/s12917-026-05584-8 42192425 PMC13393699

[B27] Tang K. Zha Y. Chen C. Lu S. Jiang X. (2025). The clinical characteristics and outcomes of anaerobic spondylodiscitis: A retrospective observational study. BMC Infect. Dis. 26, 156. doi: 10.1186/s12879-025-12445-6 41436957 PMC12837079

[B28] Walker G. K. Suyemoto M. M. Hull D. M. Gall S. Jimenez F. Chen L. R. . (2021). Genomic characterization of a nalidixic acid-resistant salmonella enteritidis strain causing persistent infections in broiler chickens. Front. Vet. Sci. 8, 725737. doi: 10.3389/fvets.2021.725737 34540936 PMC8440904

[B29] Wu H.-C. Chiu Y.-T. Wu I.-C. Liou C.-H. Cheng H.-W. Kuo S.-C. . (2025). Streamlining whole genome sequencing for clinical diagnostics with ONT technology. Sci. Rep. 15, 6270. doi: 10.1038/s41598-025-90127-8 39979452 PMC11842811

[B30] Yang P. Liu C. Zheng J. Yi J. Wu Z. Tian Y. . (2025). Rapid replacement of blaKPC variant in ST11 carbapenem-resistant and hypervirulent klebsiella pneumoniae contributed to ceftazidime/avibactam resistance during severe *in vivo* infection. J. Antimicrob. Chemother. 80, 3034–3042. doi: 10.1093/jac/dkaf337 40973133 PMC12598770

[B31] Zhao G. Lou Z. Zhu Z. Lai X. Li J. Lou X. . (2023). Epidemiological and molecular evidence of foodborne poisoning outbreak caused by enterotoxin gene cluster-harboring staphylococcus aureus of new sequence type 7591. Int. J. Infect. Diseases: IJID: Off. Publ. Int. Soc. For. Infect. Dis. 135, 132–135. doi: 10.1016/j.ijid.2023.08.005 37619662

[B32] Zhu L. Zhang C. Liang C. Peng L. Yan H. Liang X. . (2024). Molecular epidemiological characteristics of osteoarthritis-associated brucella melitensis in China: Evidence from whole-genome sequencing-based analysis. Ann. Clin. Microbiol. Antimicrob. 23, 18. doi: 10.1186/s12941-024-00671-w 38402187 PMC10893595

[B33] Zou X. Li X. He K. Song Q. Yin R. (2025). Current knowledge of vertebral osteomyelitis: A review. Eur. J. Clin. Microbiol. Infect. Diseases: Off. Publ. Eur. Soc. Clin. Microbiol. 44, 213–231. doi: 10.1007/s10096-024-04983-9 39589654

